# Observational study of patient and surgeon preoperative preparation in ten companion animal clinics in Ontario, Canada

**DOI:** 10.1186/1746-6148-9-194

**Published:** 2013-10-05

**Authors:** Maureen E C Anderson, Brittany A Foster, J Scott Weese

**Affiliations:** 1 Department of Pathobiology, University of Guelph, Guelph, Ontario N1G 2W1, Canada; 2 Department of Population Medicine, University of Guelph, Guelph, Ontario N1G 2W1, Canada; 3 Biological Sciences Department, University of New Orleans, New Orleans, LA 70148, USA

**Keywords:** Veterinary, Surgery, Disinfection, Companion animal, Surgical site antisepsis, Hand antisepsis, Video observation

## Abstract

**Background:**

Surgical site infections (SSIs) are a recognized risk of any surgical procedure in veterinary medicine. One of the keys to prevention of SSIs is reducing exposure of the surgical site to endogenous and exogenous microbes, beginning in the preoperative period. While guidelines are available for preoperative preparation procedures, there has been no objective investigation of compliance with these recommendations in veterinary practices. The objectives of this pilot study were to describe preoperative patient and surgeon preparation practices in a sample of non-equine companion animal veterinary clinics, and to determine if there were any areas that consistently did not meet current guidelines.

**Results:**

Observation of preparation practices was performed in 10 clinics over 9–14 days each using up to 3 small wireless surveillance cameras. Data were coded for 148 surgical patients, and 31 surgeons performing 190 preoperative preparations. When patient hair removal was observed, it was most commonly done using clippers (117/133, 88%), and in only one case was it performed prior to anesthetic induction. Patient contact time with soap ranged from 10-462 s (average of clinic means 75 s, average of clinic medians 67 s), and with alcohol from 3-220 s (average of clinic means 44 s, average of clinic medians 37 s). Alcohol-based hand rub (AHR) was used preoperatively in 2/10 facilities, but soap-and-water hand scrub was most commonly used at all clinics. Proximal-to-distal scrubbing was noted in 95/142 (67%) of soap-and-water scrubs. Contact time during surgeon hand preparation ranged from 7-529 s (average mean 121 s, average median 122 s) for soap-and-water and from 4-123 s (average mean 25 s, average median 19 s) for AHR. No significant changes in practices were identified over time during the observation period. Practices that did not conform to guidelines available in major companion animal surgical textbooks were commonly observed.

**Conclusions:**

Some preoperative preparation practices were relatively consistent between clinics in this study, while others were quite variable. Contact times with preoperative preparatory solutions for both patients and surgeons were often shorter than recommended. Evidence-based guidelines for these procedures in veterinary medicine should be established and implemented in order to help reduce preventable SSIs, while maintaining efficiency and cost-effectiveness.

## Background

Surgical site infections (SSIs) are common hospital-associated complications in human medicine, affecting an estimated 300 000 to 500 000 patients every year in the United States alone [1]. While the impact of SSIs is poorly quantitated in veterinary medicine, SSIs are not uncommon, with reported rates ranging from 1-18% in dogs and cats, and these infections can result in significant patient morbidity and mortality [2-4]. Hundreds of thousands of pets undergo elective and non-elective surgical procedures every year, and although the risk of SSI is low in most cases, it is never zero, and it can be much greater for some animals. Not every SSI is preventable, but many are, and the goal of perioperative infection control efforts is to reduce the preventable fraction of these infections to zero [5]. The importance of preoperative preparation of the patient and surgeon for this purpose was first reported by Joseph Lister in 1867, and by the turn of the 20th century he and others had shown that such measures could have a dramatic impact on SSI rates and ultimately patient survival [6-8]. Since then, preoperative preparation has become such a basic standard practice that published studies can only compare the impact of different techniques on various outcomes, as the absolute effect was so clearly demonstrated more than a century ago.

The focus of this study was preoperative preparation of the patient and surgeon in non-equine companion animal facilities, as it pertains to the potential for reduction of preventable SSIs. Anecdotally, these procedures vary considerably between clinics, and there has been no evaluation of practices currently in use. Basic guidelines for preoperative preparation in veterinary medicine are published in surgical textbooks [9-11], and a small number of other easily accessible sources [12], but the veterinary literature contains a relative paucity of information on the comparison of different means of surgeon and surgical site preparation. The objectives of this pilot study were to describe preoperative patient and surgeon preparation practices in a limited number of companion animal veterinary clinics, to investigate factors that may be associated with specific key practices, and to determine if there were any areas that consistently did not meet currently available guidelines. In addition, a review of preoperative preparation guidelines available in current small animal surgery textbooks was undertaken.

## Methods

A convenience sample of 10 companion animal veterinary clinics in southwestern and eastern Ontario, Canada, was recruited to participate. Each clinic was contacted directly by one of the investigators (MA or JW) by e-mail or telephone. Data collection was performed from February to August 2010. Wireless indoor video surveillance cameras (Logitech WiLife™ Indoor Video Security System, Logitech, Newark, CA) were installed by one of the investigators (MA) on day 0 to monitor each of the following areas: patient preparation table, surgical scrub sink, gowning and gloving area and surgical table in the surgical suite. Two or three cameras were used at each clinic simultaneously depending on clinic layout, routine preoperative procedures and equipment availability. The cameras were visible to staff but care was taken to position the cameras and secure their power cords to make them as discreet as possible. All indicator lights on the cameras were disabled so there were no visible signs that the cameras were on or off. Video data were recorded by powerline network on a secure, closed laptop computer kept elsewhere in the clinic in an unobtrusive location (e.g. on top of a cupboard, under a desk, on an unused shelf). Cameras were left in place for 9–14 working days (12–20 calendar days), and were motion-activated during the hours when routine surgeries were typically performed in each clinic. The cameras did not record audio data. Written consent was obtained in advance from all personnel whose images would potentially be captured on video, and participants were informed as to the general purpose of the study, but not what specific parameters were being investigated. During either the set-up or take-down site visit to each clinic, a technician or veterinarian was asked to show the investigator (MA) what products were used for preoperative preparation of the surgeon and patient; product names and active ingredients (agents) were recorded, as well as in what order the products were typically used, if applicable. This study was approved by the University of Guelph Research Ethics Board.

A video coding scheme was developed in the form of a fillable spreadsheet (Excel 2008 For Mac, Microsoft Corporation, Redmond, WA) and tested by two of the investigators (MA and BF) using the preliminary data from two clinics. The remaining videos were coded by one investigator (BF). Videos were generally scanned at 2–4 times normal speed, and then watched in real time or slow motion with repeated review as necessary to discern pertinent actions. The spreadsheet data were then imported directly into a statistical software program (STATA Intercooled 10, StatCorp LP, College Station, Texas) to perform descriptive and quantitative analyses. Preoperative patient preparation procedures (“patient preps”), including species, surgical site/procedure (abdominal, neuter, limb, other), hair removal, skin antisepsis technique, transportation to the surgical suite, draping, and performance of a “final prep” (application of a biocide after transportation to and positioning on the surgical table immediately before the procedure began) were analyzed separately from preoperative surgeon preparation procedures (“surgeon preps”), including hand antisepsis technique, gowning, gloving and surgical cap/mask use. Patient species and surgical site/procedure were also recorded for surgeon preps, unless the procedure for which the surgeon prepared was performed in an off-camera area or room, in which case these variables were recorded as unknown (this also resulted in coding of a larger number of surgeon preps than patient preps). For patient preps, contact time with soap or alcohol was measured as the time from first contact of the skin with the product to the time of first contact of the skin with any other product that would remove it (e.g. alcohol or a biocide solution/tincture). If no subsequent preparation step was observed (e.g. no subsequent step was done, animal was moved off camera, camera was blocked) the total contact time was not recorded. For surgeon preps, contact time was measured from the point the product used first came in contact with the skin to the start of rinsing (if soap and water used) or cessation of rubbing (if alcohol-based hand rub (AHR) used). If the surgeon left the field of view and did not return while still rubbing hands with AHR, no contact time was recorded.

### Statistical analysis

Outcomes evaluated using quantitative analysis were limited to contact time (with soap or alcohol for both patients preps and surgeon preps) and performance of a final prep for patients, as these outcomes could be most objectively measured and were considered critical control points in the overall preparation process. Factors included in the linear regression models (contact times) and logistic regression model (final prep) were determined *a priori* based on what data could be collected from the videos, factors thought to be the most likely to have an effect on the outcome (species, surgical site/procedure), and the need to control for clustering by clinic (initially included as a random effect in all models) and by surgeon (included as a random effect in surgeon prep models). The number of days the cameras had been present at the time of each observation was included in all models to determine if the duration of the presence of the cameras had any effect on the outcomes. The intraclass correlation coefficient (ICC) was calculated for each random effect variable in each linear model in order to assess the amount of correlation at each level, according to a standard formula for multi-level models [13]. Residuals for all models were assessed graphically using scatter plots and normal quantile plots, and outliers were further examined to ensure they were not the result of errors in data entry. For the linear models, the normality of best linear unbiased predictors (BLUPs) was assessed using normal quantile plots to evaluate model fit.

### Review of preoperative preparation guidelines in veterinary textbooks

Small animal surgery textbooks for review were selected based on accessibility and those that were anecdotally most commonly used by veterinarians in Ontario. For textbooks with multiple editions, only the most recent edition was reviewed. All relevant chapters regarding preoperative patient and surgeon preparation were examined, and the recommendations given by each textbook for pre-selected common procedures were summarized in table format.

## Results

A total of 17 clinics were approached to participate in the study. Two did not respond to phone or e-mail inquiries, three declined with no reason given, one declined stating that the clinic was too busy to participate at the time, and one declined stating that the staff were not comfortable with the use of the video cameras. The 10 clinics that agreed to participate in the study were all exclusively companion animal (non-equine) facilities, including single- and multiple-veterinarian practices, primary care and referral clinics, and clinics in both urban and suburban locations across southwestern and eastern Ontario.

In 3 clinics, a sufficient number of cameras was not available to monitor all patient and surgeon preparation areas as well as the surgical suite(s), therefore details of final preparation procedures in the surgical suite could not be recorded in all cases.

Variations in denominator values were due to procedures that were not applicable to a given patient or surgeon (e.g. if gloves were not used then glove contamination was coded as not applicable) and/or procedures that were not performed in view of the camera but may or may not have been performed off-camera (coded as not visible).

The agents contained in the products reportedly used by the participating clinics for routine patient and surgeon preps are shown in Table 1.

**Table 1:** Agents used for routine preoperative patient preparation and surgeon preparation in 10 companion animal clinics

**Agent**	**Recommended contact time**^**a**^	**Number of clinics using for:**		
		**Patient prep**	**Final patient prep**	**Surgeon prep**
70-99% isopropanol	-	8	3	-
70% isopropanol, 0.5% CH	2+ min	5	4	1
61% ethanol, 1% CH	1.5-2 min [9,11]	-	-	1
4% CH	3 min twice	4	-	9
0.1% CH (prepared in house)	-	1	-	-
2% chloroxylenol	2 min	4	-	2
0.6% chloroxylenol	Not labeled for pre-op use	-	-	1
7.5% povidone iodine	5 min	1	-	-
10% povidone iodine	Paint & dry, 5 min	2	2	-
0.2% ammonium chlorides	Not labeled for use on patients	1	1	-

CH = chlorhexidine.

^a^recommended contact time for preoperative use on surgical site or hands, as per product label.

The fit of all regression models based on graphical assessment of residuals and BLUPs was considered adequate.

### Patient preparation

A total of 148 patient preps were recorded. The number of patient preps per clinic and per surgical site/procedure is shown in Table 2.

**Table 2:** **Number of patient preps**^**a **^**recorded using video observation in 10 companion animal clinics**

		**Surgical site/procedure**				
**Clinic**	**Total patient preps**^**a**^	**Abdominal**	**Dog neuter**	**Cat neuter**	**Limb**	**Other**
1	16	8	1	4	1	2
2	30	14	4	7	1	4
3	4	1	2	0	1	0
4	13	6	3	3	0	1
5	20	15	0	4	1	0
6	3	0	0	3	0	0
7	9	4	3	1	0	1
8	36	7	0	0	24	5
9	10	1	4	3	1	1
10	7	2	2	0	1	2
Total	148	58	19	25	30	16

^a^preoperative patient preparation procedures.

All clinics routinely used clipping for hair removal (117/148 cases, 79%), except for cat neuters for which hair was plucked in 16/25 (64%) cases at five clinics. A razor (shaving) was used once following clipping for an aural surgery. In 10 cases hair had already been removed before the animal was in view of the camera, and in 5 cases no hair removal was performed (4 declaws, 1 small lumpectomy). One clinic regularly clipped and performed all skin preparation steps on patients in the surgical suite. All other clinics clipped and performed at least one skin preparation step prior to moving the patient to the surgical suite. Hair removal prior to anesthetic induction was observed in one patient that appeared heavily sedated.

Application of a soap-and-water scrub during patient prep was observed in all clinics. Soap-and-water scrub was applied with a “back and forth” technique in 9/10 clinics, and with a “circular” technique in 7/10 clinics, but in 4 of the latter clinics “back and forth” was still used more often than the “circular” technique. Application of an alcohol step was observed in 9/10 clinics; this was performed in a “centre to edge” or “concentric circles” pattern in 6/9 clinics, whereas a “top to bottom” linear technique was used in 8/9 clinics. Of the 8 clinics in which another antiseptic step was observed (instead of or in addition to alcohol), 4 used “centre to edge” or “concentric circles”, whereas 6 used “top to bottom”, which was the most common technique used (40/83 observed applications, 48%). Use of multiple techniques was observed at most clinics. Reuse of the same surface of a disposable gauze square after a single contact with the patient’s skin was frequent for application of alcohol (59/62, 95%) and antiseptic (53/60, 88%).

Non-sterile contact (e.g. by clothing, unclipped limbs, drape surfaces previously in contact with unprepared sites) with the surgical site during transportation of the animal to the surgical suite was seen in 39/107 (36%) cases in 7/8 clinics in which this procedure was observed, but non-sterile contact may have actually occurred in a higher proportion of patients than this, as it was not possible to track all animals during the entire transportation procedure due to the fixed camera positions. A total of 27/148 (18%) animals from 9 clinics had their procedures performed in the patient preparation area instead of the surgical suite; these included 22 (81%) cat neuters, 3 procedures on digits (including declaws), drainage of an aural hematoma and debridement of two preexistent surgical incision sites on the same animal. Out of 64 procedures for which a final preparation step in surgery was observed, non-sterile contact with the surgical site was observed during 7 (11%). A multivariable mixed-effects logistic regression model for performance of a final prep was constructed, including clinic as a random effect and surgical site and species as fixed effects. The number of days the cameras were present in the clinic was also initially included in the model, but because it was not linearly associated with the outcome, the variable was categorized and found to have no significant effect (partial F-test p = 0.756) and was therefore dropped. According to the final model, final prep in surgery was significantly less likely to be performed for neuters compared to abdominal surgeries (odds ratio (OR) 0.13, p = 0.013, 95% confidence interval (CI) 0.02-0.65), and was more likely to be performed for dogs compared to cats (OR 5.2, p = 0.005, 95%CI 1.62-16.57).

Contact time with soap (Table 3) was recorded for 105 patient preps in 9/10 clinics, and ranged from 10–462 s. The average of the mean contact time for each clinic was 75 s, and the average of the median contact time was 67 s. Contact time with alcohol (Table 3) was recorded for 37 patient preps in 8/10 clinics, and ranged from 3–220 s, with the average of mean contact times being 44 s and the average of median contact times being 37 s. A multivariable mixed-effects linear regression model including clinic as a random effect, surgical site/procedure, species and the number of days the cameras were present in the clinic as fixed effects showed that there was no significant difference in soap contact time between species (p = 0.511) or surgical site/procedure (partial F-test p = 0.145), and the number of days the cameras were present in the clinic also had no significant effect (p = 0.075). The ICC for clinics was 0.36. A similar model constructed with the same variables showed that alcohol contact time was significantly longer for dogs compared to cats (26 s, p = 0.039, 95%CI 1–51), but surgical site/procedure had no significant effect (partial F-test p = 0.314). The number of days the cameras were present had no significant effect (p = 0.997), and the ICC for clinics was 0.35.

**Table 3:** **Product contact times**^**a **^**during patient preps**^**b **^**recorded using video observation in 10 companion animal clinics**

	**Soap-and-water patient prep**^**b **^**step**				**Alcohol patient prep**^**b **^**step**			
**Clinic**	**n**	**Mean contact time (s)**	**Median contact time (s)**	**Range (s)**	**n**	**Mean contact time (s)**	**Median contact time (s)**	**Range (s)**
1	14	48	46	18 - 150	11	32	22	14 - 66
2	30	82	70	18 - 247	3	106	60	28 - 220
3	4	59	54	46 - 82	0	-	-	-
4	12	52	38	20 - 178	1	30	30	-
5	3	24	30	10 - 33	6	26	28	3 - 49
6	3	54	50	30 - 81	3	38	39	33 - 43
7	0	-	-	-	0	-	-	-
8	25	107	72	34 - 462	2	70	70	50 - 89
9	8	29	29	18 - 47	5	10	11	5 -13
10	6	219	214	140 - 369	6	38	38	6 - 76
Total	105	75^c^	67^d^	10 - 462	37	44^c^	37^d^	3 - 220
		80^e^	54^f^			37^e^	30^f^	

^a^contact times were only recorded for those preparation steps which were followed by an another preparation step (e.g. alcohol, antiseptic).

^b^preoperative patient preparation procedures.

^c^average of the means for each clinic.

^d^average of the medians for each clinic.

^e^overall mean.

^f^overall median.

### Surgeon preparation

A total of 190 surgeon preps performed by 31 individual surgeons were recorded. The number of surgeons observed and the number and type of surgeon preps performed at each clinic are shown in Table 4. The mean number of surgeon preps observed per surgeon was 6 (range 1–21, median 4).

**Table 4:** **Product contact times during surgeon preps**^**a **^**recorded using video observation in 10 companion animal clinics**

			**Soap & water surgeon preps**^**a**^				**Alcohol-based surgeon preps**^**a**^			
**Clinic**	**No. of surgeons**	**Total surgeon preps**	**n**	**Mean contact time (s)**	**Median contact time (s)**	**Range (s)**	**n**	**Mean contact time (s)**	**Median contact time (s)**	**Range (s)**
1	4	17^b^	8	41	34	22 - 90	6	12	10	4 - 25
2	2	30^c^	29	40	34	11 - 79	-	-	-	-
3	1	4	4	154	140	134 - 200	-	-	-	-
4	2	8	8	145	142	68 - 217	-	-	-	-
5	4	23	23	119	134	7 - 216	-	-	-	-
6	2	3	3	127	139	89 - 153	-	-	-	-
7	2	8^d^	7	210	200	96 - 402	-	-	-	-
8	10	81^e^	46	261	274	28 - 529	31	38	27	11 - 123
9	1	9	9	25	29	10 - 35	-	-	-	-
10	3	7	7	88	93	41 - 120	-	-	-	-
Total	31	190	144	121^f^	122^g^	7 - 529	37	25^f^	19^g^	4 - 123
				144^h^	124^i^			34^h^	25^i^	

^a^preoperative surgeon preparation procedures (scrub or rub of hands +/− arms).

^b^2 alcohol preps were started but not completed in view of the camera, surgeon gloved without prepping for one procedure (all cat neuters).

^c^hands rinsed with water only for 1 procedure (cat neuter).

^d^regloving alone performed for one procedure without additional prep.

^e^3 soap & water preps were started but not completed in view of the camera, regloving alone performed for one procedure without additional prep.

^f^average of the means for each clinic.

^g^average of the medians for each clinic.

^h^overall mean.

^i^overall median.

Contact time with soap-and-water was recorded for 144 surgeon preps in 10/10 clinics, and ranged from 7–529 s. The average of the mean contact time for each clinic was 121 s, and the average of the median contact time was 122 s. The 11 shortest soap-and-water scrubs (range 7–19 s) all preceded cat neuters. For cat neuters, scrub duration ranged from none (following a previous surgery) to 153 s (mean 38 s, n = 23) at 6/10 clinics, while duration of scrubs for dog neuters ranged from 25–402 s (mean 114 s, n = 18) at 7/10 clinics. The mean duration of scrubs prior to abdominal surgeries ranged from 20–313 s (mean 114 s, n = 52) at 9/10 clinics. Scrubs done with a bristle sponge ranged in length from 17–529 s (mean 166 s, n = 121) at 9/10 clinics; the remaining scrubs were performed without any brush or sponge at 4/10 clinics and ranged in length from 7–90 s (mean 28 s, n = 23). Proximal to distal scrubbing (e.g. scrubbing from arms to hands instead of hands to arms) was noted in 95/142 (67%) soap-and-water surgeon preps at 9/10 clinics, for 25/31 (81%) surgeons.

Alcohol-based hand rub was used for preoperative hand antisepsis by 9 surgeons in 2/10 facilities, but even at these clinics soap-and-water scrub was more commonly used (see Table 4). Only one of the two clinics used an AHR that was labeled for preoperative hand antisepsis (alcohol-based surgical hand rub, ASHR) as opposed to an AHR intended for routine hand hygiene, although in the latter clinic use of the AHR was always preceded by a basic hand wash with 4% chlorhexidine soap. Contact time with alcohol was recorded for 37 procedures and ranged from 4–123 s. The average of the mean contact time for each clinic was 25 s, and the average of the median contact time was 19 s. Use of AHR was not observed prior to any neuters. Other notable practices that were observed during surgeon prep included two individuals shaking their hands to dry (instead of rubbing to dry) when using AHR [7].

The mixed-effects linear regression model for surgeon prep soap-and-water contact time included clinic and surgeon as random effects, and surgical site/procedure, species and the number of days the cameras were present in the clinic as fixed effects. Surgeon preps for which the surgical site/procedure and species were unknown were excluded (n = 19). The model showed no significant difference in contact time for cats compared to dogs (p = 0.130). Soap-and-water contact time was significantly longer for limb procedures compared to abdominal procedures, neuters and all “other” surgical sites (55 s, p = 0.011, 95%CI 13–98; 77 s, p = 0.001, 95%CI 30–124; 95 s, p < 0.001, 95%CI 43–146 respectively) and significantly longer for abdominal procedures compared to all “other” surgical sites (40 s, p = 0.032, 95%CI 3–76). The ICC for clinics was 0.52 and for surgeons was 0.74. The number of days the cameras were present in the clinic had no significant effect on soap-and-water contact time (p = 0.380).

Because AHR was only used at two clinics, the model constructed for AHR contact time included surgeon as a random effect, and clinic, surgical site/procedure, species and number of days the cameras were in place all as fixed effects. Surgeon preps for which the surgical site/procedure and species were unknown were excluded (n = 11). The only significant factor in the model was surgeon (p = 0.0227 for likelihood ratio test vs linear regression model without random effects); however, because the model included only 26 observations it had relatively low power to detect differences in the other variables. The ICC for surgeons was 0.52.

Surgical gloves were used for 176/188 (94%) observed procedures. For 65 (35%) of these the gloving technique used was not seen. Open gloving was used in 61 (32%) cases in 8/10 clinics by 17 surgeons. Closed gloving was used in 50 (27%) cases in 7/10 clinics by 16 surgeons, but poor technique including exposure of fingers or contact with a non-sterile cuff (as when regloving) was observed in 21 of these (5 surgeons) [9]. Other contact with the outside of the gloves while donning was seen in 5 cases (4 surgeons) in which the view was unobstructed. Of 12 (6%) surgeries for which gloves were not used, 10 were cat neuters performed at 3 clinics, and 2 were cat declaws performed at 2 clinics. For two procedures use of gloves could not be confirmed due to camera obstruction.

A gown was used in a total of 133/190 (70%) procedures, including 42/63 (67%) abdominal procedures, 11/41 (27%) neuters and 38/39 (97%) limb procedures. In 9 cases, including 2 abdominal procedures at 2 clinics, the gown used had clearly been in contact with non-sterile surfaces or had been used for a previous surgery. Six (19%) surgeons at 3 clinics did not wear a gown for at least one abdominal procedure. A mask was used in 175/190 (92%) procedures, including all abdominal and limb procedures and 26/41 (63%) neuters. A cap was also used in all but 8 cases (involving 2 surgeons) when a mask was used. All procedures for which a mask was not worn were cat neuters, performed at 5 clinics.

### Review of preoperative preparation guidelines in veterinary textbooks

The most recent editions of two major small animal surgery textbooks [9,10] that were available at the time of the study were reviewed to compare their recommendations, as well as one of the most recently published small animal surgery textbooks [11]. The results of the comparison are shown in Table 5. Of note:

•All three textbooks primarily recommended the use of chlorhexidine (CH) or povidone iodine (PI) (either aqueous or tincture) for patient preparation, and two stated that other agents are not recommended, as CH and PI are superior.

•When specified, the recommended agent contact time for patient prep was consistently at least 2 minutes, with the exception of the most recent textbook which also discussed newer one-step prep agents, but emphasized that manufacturer’s instructions should always be followed for any specific product.

•Likewise, recommended contact times for soap-and-water surgeon prep were universally at least 2 minutes, and up to 7 minutes. Recommended contact times for ASHR were 1.5-2 minutes, or as per manufacturer’s instructions.

**Table 5:** Comparison of recommendations for preoperative preparation from three major small animal surgery textbooks

**Recommendation**	**Slatter 2002**[10]	**Fossum 2007**[9]	**Tobias & Johnston 2012**[11]
Clip hair right before surgery	√	√	(√)^d^
Patient prep with CH or PI	√	(√)^c^	√
Patient prep with any other agent	(√)^a^	X	X
Contact time for patient prep (depending on agent)	>2 min	2-3 min	0.5-2 min ^e^
Apply antiseptic in a center-to-periphery circular pattern	√	√	-
Surgeon prep with CH or PI scrub	√	√	(√)^f^
Surgeon prep with ASHR	(√)^b^	√	√
Surgeon prep with any other agent	-	-	-
Contact time for surgeon prep (soap & water, first scrub of day)	5 min	5-7 min	-
Contact time for surgeon prep (soap & water, any scrub)	2-5 min	2-3 min	-
Contact time for surgeon prep (ASHR)	-	1.5-2 min	-^e^
Surgeon prep technique described	√	√	-^e^
Wear mask during surgery	√	√	(√)^g^
Wear headcover during surgery	√	√	(√)^h^
Wear gown during surgery	√	√	√
Gowning technique described	√	√	-
Gloving technique(s) described	√	√	-
Notes	Formerly required ACVS reading	Current required ACVS reading	Current required ACVS reading, not available at time of study

√ = recommended, (√) = recommended with caveats, X = not recommended, - = no specific recommendation/not described, prep = preparation, CH = chlorhexidine, PI = povidone iodine, ASHR = alcohol-based surgical hand rub, ACVS = American College of Veterinary Surgeons.

^a^use of chloroxylenol described, but not recommended for use in cats.

^b^ASHR reportedly superior to CH or PI but not as commonly used.

^c^CH or PI in alcohol preferred.

^d^evidence is inconsistent.

^e^follow manufacturer’s recommendation for specific product.

^f^both agents significantly decrease bacterial load on hands, but ASHR is preferred.

^g^mask use helps prevent direct contamination during talking, no effect on environmental bacterial load.

^h^use of headcovers controversial, but hair carries higher bacterial load so makes sense to cover.

While all three textbooks favourably discussed the use of ASHR, the more recent the publication, the more the use of ASHR was promoted over soap-and-water scrub.

## Discussion

Microbial contamination of a surgical site is a prerequisite for all SSIs, therefore one of the primary goals of SSI prevention practices is to reduce the spread of microbes to and at the surgical site, beginning in the preoperative period. At no time are the tissues more susceptible to invasion than during the surgical procedure itself, which makes preoperative preparation of both the patient and surgeon critical for SSI prevention.

The goal of preoperative patient skin preparation is to reduce the transient and to some extent the resident microbiota of the skin as rapidly and atraumatically as possible, and to prevent short-term (i.e. hours) rebound growth of opportunistic bacterial pathogens. This helps to protect sterile sites from invasion by the patient’s own endogenous flora, which is critical as the majority of surgical site infections are caused by bacteria that are already carried by the patient at the time of surgery [14]. In this study, some preoperative patient preparation practices were relatively consistent between clinics, while others were not. Hair removal was routinely performed using clippers in all clinics after patient induction, and the majority of clinics performed hair removal and at least one preparation step outside the surgical suite, which is consistent with current recommendations and evidence for reducing SSI risk [9-11,15]. The techniques with which skin preparation solutions were applied varied considerably between and within clinics, which may reflect a lack of standardized protocols, with subsequent day-to-day differences in staff performing these procedures. A number of different application techniques are used for skin antiseptics in human medicine as well, and currently there is insufficient evidence to recommend one technique over another [16]. Nonetheless, it is typically recommended that application is done in a “cleanest-to-dirtiest” pattern of some kind, starting with a clean applicator (e.g. gauze) at the centre of the prepared area (i.e. the incision site) and moving toward the periphery, usually in concentric circles [9,10,14,17]. This is based on the fundamental principles of aseptic technique, as it helps to avoid contaminants from unprepared areas of skin being dragged onto cleaner areas of skin on the applicator itself. Even so, other techniques that do not conform to this principle (e.g. “back and forth” and “top to bottom”) were commonly observed. These practices could potentially be improved through basic staff education and training.

In human patients, skin preparation in the immediate preoperative period involves application of antiseptic in either a single or multiple steps [16]. However, the comparatively dense hair coats of animals and other species-specific factors may lead to very different skin conditions and levels of contamination in veterinary patients, therefore a multi-step process is typically used for patient preparation, as was observed in all clinics in this study. However, it is unknown which step of the preparation procedure is the most critical for reducing the microbiota. Until more information is available, it seems prudent to the authors to ensure that each product employed in the protocol is used according to the manufacturer’s recommendations in order to obtain the most benefit. Contact times with skin preparation products were often far shorter than those recommended by the manufacturer. These product-specific recommendations are established to ensure a minimum reduction in bacterial numbers on treated skin, and if they are not observed the product is less likely to be effective. In only one clinic was the mean contact time with antiseptic soap during patient preparation greater than the minimum recommended contact time for the product used (2–5 minutes, see Table 3). Ensuring standard clinic protocols for application of these agents are followed could help to improve contact times, as well as basic application technique.

The two most effective and commonly used antiseptic agents for surgical site preparation are PI and CH in either alcohol or aqueous solutions [5,11,14,16,18]. Despite numerous studies in the human medical literature, there is still no consensus on which agent is best for reducing SSI rates, in part due to the variety of preparations available in either an alcohol or aqueous base [16]. For example, a recently published randomized control trial found a lower SSI rate in patients prepared using CH in alcohol versus aqueous PI, but the trial was criticized for not comparing CH in alcohol to PI in alcohol [1]. Use of other agents is generally not recommended because they are less effective than CH or PI [9,11]. Although in the current study 6/10 clinics reported using a CH or PI product for patient preparation, 4/10 clinics used products containing other agents (chloroxylenol or an ammonium chloride compound) instead. The reasons for selecting these other products were not investigated.

The goal of preoperative hand antisepsis is similar to that of patient skin preparation, and ultimately helps to protect the patient’s tissues from invasion by exogenous bacteria from the surgeon’s hands. Rapid and atraumatic methods of reducing the microbiota of the skin are particularly important for surgeons who may need to perform the procedure frequently, in order to prevent skin damage that can lead to increased carriage of bacteria on the hands [11]. However, adequate contact time with hand preparation agents, as for patient preparation agents, is crucial to achieve the required reduction of bacterial numbers. Although recommended contact times with traditional preoperative hand scrub agents have decreased from 10 minutes or more to as little as 2 minutes [7,9,10,19], in this study contact time (and technique) for soap-and-water surgeon preparation were highly variable, with almost half failing to meet the minimum recommended 2-minute scrub time.

Alcohol-based surgical hand rubs have been the recommended method of choice for surgeon hand antisepsis in human medicine for some time [20]. These agents have a rapid-kill effect due to their alcohol content, and often contain another antiseptic agent such as CH that remains on the skin and provides a more prolonged antimicrobial effect [7,11,21]. They take less time to apply, and perhaps most importantly are the least traumatic means of reducing the microbial flora of the hands, particularly with repeated use, when compared to traditional soap-and-water scrub [7]. In two clinical trials comparing traditional hand scrub to an ASHR that measured SSIs as the primary outcome, ASHR was found to perform equally well, the ASHR protocol was better accepted by personnel [22], and in a cost analysis was 40% less expensive per application [23]. The use of ASHR also reduces waste (sponges, drying towels), saves a considerable amount of water (traditional scrubs typically require approximately 20 L of water per person [7]), and negates the risk of recontamination of the hands in the case of potentially contaminated water faucets/sinks or questionable water quality.

Despite the widespread acceptance of ASHRs for preoperative hand antisepsis in human medicine, and the body of evidence available that supports their use, veterinarians have been slow to adopt this technique. A survey of board-certified veterinary surgeons in North America and Europe in 2009 found that 80% still used antiseptic soap-and-water preoperative scrub, and of these 81% used chlorhexidine-based scrub [24]. This may be due to the perception that veterinarians’ hands may be more heavily soiled in the clinic environment because they handle animals and therefore need to be scrubbed [25], failure to communicate information about ASHRs to veterinarians as a result of the lack of information on this topic published in the veterinary literature, or simply resistance to change. Recently Verwilghen et al. [26] evaluated the efficacy of ASHR versus soap-and-water scrub in a veterinary context, and found the ASHR to have an equal or better immediate and sustained antiseptic effect, and concluded that ASHR could be an effective alternative for veterinary preoperative hand asepsis. In the current study, alcohol-based agents were infrequently used for surgeon preparation compared to soap-and-water, and in 50% of cases total contact time was 25 s or less, which does not meet the recommended contact time of 1.5-2 minutes for preoperative use of such agents [7,9,11]. Furthermore, one clinic used an AHR not labeled for preoperative use in the place of ASHR. The effectiveness of AHR intended for routine hand hygiene for preoperative hand antisepsis is unknown, and its use in this manner should be avoided given that approved ASHR products are readily available. More training and information about the use of ASHRs needs to be provided to veterinarians.

The use of sterile surgical gloves is also an important component of aseptic technique that likely contributes significantly to the prevention of SSIs by providing an effective barrier between the microbiota of the surgeon’s hands and the surgical site. Sterile gloves were used by surgeons for the majority of procedures observed in this study, although potential contamination of the outer sterile surface of gloves was seen occasionally. However, glove use does not negate the need for proper preoperative hand antisepsis. The accepted quality control limit for defects in medical gloves large enough to leak water is 1.5% [27]. In one study of glove punctures in orthopedic surgery, perforations were found in 2/200 (1%) of unused control gloves [28]. Furthermore, glove punctures have been reported to occur in up to 34-43% of human surgical procedures [29-31]. In a study performed at two companion animal hospitals, glove punctures were found in one or both gloves in 148/382 (38.7%) glove pairs, and personnel were typically unable to predict whether or not a glove defect was present at the end of procedures [32]. Proper hand antisepsis prior to gloving for surgery therefore remains critical.

Intraclass correlation coefficients are useful for estimating the correlation in outcome measures within clusters of mixed models (i.e. those including random effects)[13]. In this study, the ICCs estimate the correlation in contact times for surgeon and patient preparations within each clinic, and for surgeon preparations within each surgeon within a clinic. The ICC for clinics for contact time with soap-and-water and alcohol in patient preps were relatively low (0.35-0.36), which suggests considerable variability in protocols used within each clinic, and is consistent with the wide range of contact times and techniques observed. The ICC for surgeons for contact time with soap-and-water was higher (0.74), suggesting that each surgeon’s individual routine was relatively consistent. Surgeon was the only significant factor in the model for AHR contact time, but the ICC was 0.52, indicating that AHR contact time between preps for each surgeon was likely only moderately consistent.

Due to the limited amount of information on preoperative preparation of the patient and surgeon in the veterinary literature, veterinary surgery textbooks are one of the most likely sources of information regarding these procedures for veterinarians in private practice. Based on the results of this study, including use of agents other than CH and PI for preoperative preparation, frequently short contact times with preparation agents and relatively infrequent use of ASHR, it seems the majority of individuals observed were unaware of the guidelines in currently available companion animal surgery textbooks, or chose not to follow them during preoperative preparation for any number of reasons (e.g. personal preference, time constraints, habit, lack of perceived adverse events from alternate methods).

Any observational study is potentially subject to bias due to Hawthorne effects, whereby individuals may modify their behavior (intentionally or unintentionally) due to the knowledge that they are being observed [33]. The use of video observation with discreet webcams over days to weeks was intended to help reduce or eliminate Hawthorne effects in this study, compared to direct observation with an observer present in the room, which would be considerably more intrusive and serve as a constant reminder of the ongoing study. If the presence of the cameras resulted in altered behavior among study participants with respect to the procedures observed, one might expect an initial artificial increase in contact times with preparation agents, due to increased focus on these activities, followed by a decrease over time as individuals became acclimatized to the equipment and returned to their typical routine [34]. Such a pattern was not apparent here, suggesting that the cameras had little impact on participant behaviour, although a static effect throughout the recording period cannot be ruled out. Based on informal verbal feedback received, the cameras seemed to be well tolerated by staff overall, and this system could be useful for future observational studies in small companion animal clinics in which direct observation of practices is not feasible.

The various limitations of this pilot study must be taken into account when interpreting the results. The sample size was small, yet covered a wide range of practice types with a variety of caseloads in terms of both type and quantity of procedures, so the power for some comparisons was quite low. Potential volunteer bias must also be considered, as clinics with more interest in improving preoperative preparation practices, or that already felt more comfortable with the quality of their practices, may have been more willing to participate. Although the use of the camera monitoring system likely provided considerably less biased and a larger quantity of data compared to direct observation, the fixed camera positions and their limited field of view resulted in incomplete data sets for many of the procedures observed. Contact time for preparation steps that were not followed by an additional preparation step (including the final preparation step) were not recorded because this information could not be captured reliably in many cases, due to the inability to determine when alcohol or alcohol-based agents had fully dried, lack of a camera in the surgical suite, or obstruction of the camera by personnel during draping and at the start of surgery that precluded determination of the time of the initial incision. In future studies, if a similar video observation system is employed, recommendations include pre-visits to all participating clinics to determine the number of cameras required in advance (to ensure an adequate number is available), and improved positioning of cameras, particularly in the surgical suite, to help reduce the risk of visual obstruction. Cameras should be placed as high up as possible or even on the ceiling (e.g. above the surgery table). Ceiling mounting of the camera system was used in this study without complications in several clinics, but was not possible in other cases due to constraints regarding power outlet locations, cable length, visual field of the cameras and concerns regarding obstruction by overhead surgical lights. Pre-visiting the clinics would also allow more time for consultation with the staff regarding optimal camera placement, and thereby increase efficiency of set up when the cameras are put in place, thus further minimizing disruption to clinic workflow.

## Conclusions

This study provides an interesting glimpse of the variety of practices being employed currently in different clinics during preoperative preparation of patients and surgeons. It brings to light a number of issues, including the need for proper use of ASHR by surgeons and other skin antiseptics for patient preparation according to manufacturers’ instructions, that can potentially be addressed through education and increasing awareness. More evidence-based guidelines for patient and surgeon preoperative preparation, ideally based on veterinary-specific studies, need to be established and implemented to help improve the standard of veterinary care and reduce preventable SSIs, while maintaining efficiency and cost-effectiveness. The camera system used had little detectable effect on the behavior of participants, and could be useful for performing similar field-based observational studies in the future.

## Abbreviations

SSI: Surgical site infection; AHR: Alcohol-based hand rub; ICC: Intraclass correlation coefficient; BLUP: Best linear unbiased predictor; OR: Odds ratio; CI: Confidence interval; ASHR: Alcohol-based surgical hand rub; CH: Chlorhexidine; PI: Povidone iodine.

## Competing interests

The authors declare that they have no competing interests.

## Authors’ contributions

MA and JW designed the study. On-site data collection (including camera set up) was performed by MA and BF. MA and BF designed and tested the video coding scheme, and videos were then coded by BF. All data cleaning and analysis was performed by MA. The manuscript was drafted by MA and revised by JW and BF. All authors read and approved the final manuscript.
